# Blood group A: a risk factor for heart rupture after acute myocardial infarction

**DOI:** 10.1186/s12872-020-01756-y

**Published:** 2020-11-03

**Authors:** Yuan Fu, Mulei Chen, Hao Sun, Zongsheng Guo, Yuanfeng Gao, Xinchun Yang, Kuibao Li, Lefeng Wang

**Affiliations:** Department of Cardiology, Chaoyang Hospital, Capital Medical University, Beijing, China

**Keywords:** ABO blood groups, Coronary artery disease, Heart rupture, Acute myocardial infarction, Percutaneous coronary intervention

## Abstract

**Introduction:**

Studies have been performed to identify the association between ABO blood groups and coronary artery disease. However, data is scarce about the impact of ABO blood groups on heart rupture (HR) after acute myocardial infarction (AMI).

**Methods:**

We conducted a retrospective case–control study that included 61 consecutive patients with HR after AMI during a period from 1 January 2012 to 1 December 2019. The controls included 600 patients who were selected randomly from 8143 AMI patients without HR in a ratio of 1:10. Univariate and multivariate logistic regression analysis were used to identify the association between ABO blood groups and HR.

**Results:**

Patients with blood group A had a greater risk of HR after AMI than those with non-A blood groups (12.35% vs 7.42%, *P* < 0.001). After adjusting for age, gender, heart rate at admission, body mass index (BMI), and systolic blood pressure (SBP), blood group A was independently related to the increased risk of HR after AMI (OR = 2.781, 95% CI 1.174–7.198, *P* = 0.035), and remained as an independent risk factor of HR after AMI in different multivariate regression models.

**Conclusion:**

Blood group A is significantly associated with increased HR risk after AMI.

## Introduction

Heart rupture (HR) was one of the fatal complications after acute myocardial infarction (AMI) though its incidence decreased dramatically in reperfusion era nowadays [1–3]. HR was specified as free wall rupture (FWR), ventricular septal rupture (VSR) and papillary muscle rupture (PMR). In the pre-perfusion time, the incidence of FWR was about 2–6%, accounting for up to 30% of the in-hospital death after AMI [3–5]. VSR happened in approximately 1–3% AMI population before the reperfusion time, with 45% and 90% death rates each for surgical and conservative treatment [6–8]. PMR often causes mitral regurgitation (MR) and present in < 1% of AMI patients who undergo early revascularization according to recent data [9]. Several previous studies have verified the association between blood group A and the increased risk of vascular diseases including coronary artery disease (CAD) [10–15]. Non-O blood groups were also determined to be a significant prognostic indicator of poor prognosis in AMI patients [16–18]. However, there is scarce or even no data about the impact of ABO blood groups on the risk of HR after AMI. Therefore, we conducted a retrospective case–control study to investigate whether there is a potential connection between ABO blood groups and the risk of HR after AMI.

## Methods

### Patient population and study design

**Fig. 1:**
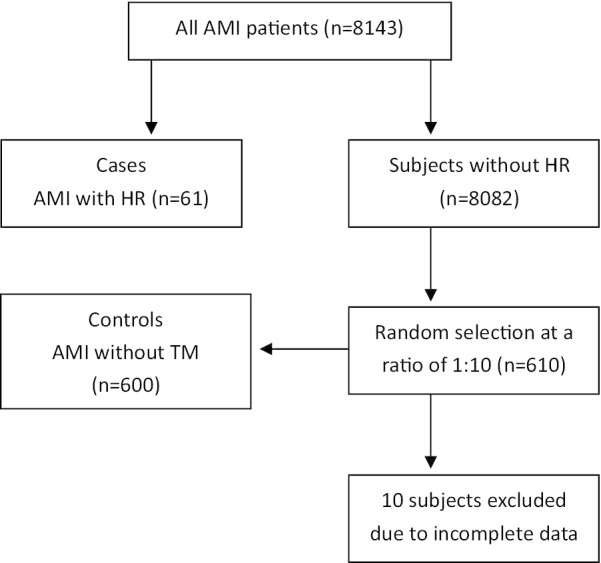
A schematic diagram of the selection of cases and controls

We retrospectively analyzed 61 consecutive patients with HR after AMI referred to Beijing Chao-Yang Hospital from 1 January 2012 to 31 December 2019. The controls included 600 patients who were selected randomly from 8143 AMI patients without HR in a ratio of 1:10 (n = 610 after excluding 10 cases with an incomplete record, Fig. 1). HR was specified as FWR, VSR and PMR.

AMI was classified as ST-segment elevation myocardial infarction (STEMI) and non- ST-segment elevation myocardial infarction (NSTEMI), the diagnostic criteria refer to our previous study [19].

FWR was defined as: (1) echo-free space can be seen on echocardiography in patients with sudden cardiogenic shock, low blood pressure or indistinct consciousness; (2) Sudden cardiac shock, low blood pressure or indistinct consciousness that associated with massive pericardial effusion confirmed by pericardiocentesis [20]. VSR was characterized by: (1) abnormal physical examination findings such as cardiac systolic murmur and cardiac tremor; (2) Ventricular septal discontinuity can be seen on echocardiography [21]. The diagnostic criteria of PMR were as follows: (1) abnormal physical examination findings such as new systolic murmur; (2) Echocardiography shows a mobile mass in either the left atrium or ventricle; (3) flail or ruptured chordae with an abnormal-looking papillary muscle [9].

### Data collection

#### Anthropometric measurements and data collection

The demographics, medical and family history, height, weight, status of medications and smoking data were collected upon admission. Estimated glomerular filtration rate (eGFR) was calculated by using Modification of Diet in Renal Disease (MDRD) formula (Chinese version) [22].

The Global Registry of Acute Coronary Events risk score (GRACE RS) is developed for risk stratification in acute coronary syndromes (ACS) patients. It is calculated from several variables: age, history of myocardial infarction, history of heart failure, systolic blood pressure (SBP), heart rate and serum creatinine level at admission, ST-segment depression, elevated myocardial necrosis markers or enzymes, and lack of percutaneous coronary revascularization during admission [23–25].

#### Laboratory parameters

Blood samples were collected in the emergency room before any therapies and analyzed by Dimension RxL Max™ automated analyzer (Dimension, USA). Automatic analyzer Hitachi 7600 (Hitachi, Japan) was used for biochemical variables measurement. All parameters were tested by using blood serum.

### Statistical analysis

All statistical analyses were conducted using SPSS 24.0 (IBM Corp, Armonk, NY). Kolmogorov–Smirnov test was used to test the normal distribution of continuous variables. Normally-distributed data are expressed as mean ± SD, and analyzed by Student's t-test. Non normally-distributed variables are presented as median (interquartile range), and analyzed by Mann–Whitney U test. Dichotomous variables were presented as frequencies and percentages, analyzed with Pearson's chi-squared test. The analysis of variance (ANOVA) test was used to examine the distribution of HR events in each blood group. Univariable analysis was used to identify the risk factors for HR. The potential association between ABO blood groups and HR after AMI was identified by multivariate logistic regression analysis. A 2-sided *P* < 0.05 was considered statistically significant.

## Results

### General characteristics

A total of 661 AMI patients (68.53% male) were included in data analyses (Table 1). Selection of all participants is shown in Fig. 1. 61 patients developed HR (0.75%) after AMI: 40 FWR (0.49%), 15 VSR (0.18%) and 6 PMR (0.07%). The mean observational time of HR after AMI was 2.72 days (VSR = 3.22 days, FWR = 2.57 days, PMR = 1.49 days). 21 FWR (52.5%), 7 VSR (46.67%) and 4 PMR (66.67%) developed within 24 h after symptoms onset (Table 2). 5 FWR (12.5%), 4 VSR (26.67%) and 1 PMR (16.67%) occurred before admission. 38 FWR (95%), 10 VSR (66.67%) and 2 PMR (33.33%) died during hospitalization. 437 AMI patients (66.11%) received primary percutaneous coronary intervention (pPCI) treatment and no patients received thrombolytic therapy or emergency coronary artery bypass grafting (CABG). Baseline characteristics of relevant patients are shown in Table 1. Compared with non-HR patients, HR patients presented more frequently with older age, female, longer time from symptom onset to admission, blood group A, higher HR at admission, KILLIP class, brain natriuretic peptide (BNP), white blood cell (WBC), erythrocyte sedimentation rate (ESR), CTNI, creatine kinase MB (CK-MB), the GRACE RS and in-hospital mortality (*P* < 0.05 versus non-HR patients for all measures). HR patients had significantly lower BMI, red blood cell (RBC), hemoglobin (Hb), left ventricular ejection fraction (LVEF), estimated glomerular filtration rate (eGFR), and less possible to receive pPCI treatment (*P* < 0.05 versus non-HR patients for all measures).

**Table 1:** Baseline characteristics of the study population

Variables	FWR patients (n = 40)	VSR patients (n = 15)	PMR patients (n = 6)	HR patients (n = 61)	Non-HR patients (n = 600)	*P* value (HR vs Non-HR)
Age, years	76.59 ± 5.48	74.36 ± 4.24	74.18 ± 5.02	75.56 ± 5.12	66.18 ± 6.63	< 0.001
Male, n (%)	16 (40)	7 (46.67)	3 (50)	26 (42.62)	427 (71.17)	< 0.001
HT, n (%)	27 (67.5)	9 (60)	3 (50)	39 (63.93)	420 (70)	0.571
DM, n (%)	9 (22.5)	4 (26.67)	2 (33.33)	15 (24.59)	145 (24.17)	0.433
CHF, n (%)	2 (5)	1 (6.67)	1 (16.67)	4 (6.56)	25 (4.17)	0.81
History of MI, n (%)	3 (7.5)	3 (20)	1 (16.67)	7 (11.48)	65 (10.83)	0.917
History of CAD, n (%)	6 (15)	4 (26.67)	2 (33.33)	12 (19.67)	115 (19.17)	0.743
History of PCI, n (%)	3 (7.5)	2 (13.33)	1 (16.67)	6 (9.84)	83 (13.83)	0.383
History of CABG, n (%)	1 (2.5)	1 (6.67)	0 (0)	2 (3.28)	13 (2.17)	0.419
Current smoker, n (%)	21 (52.5)	8 (53.33)	3 (50)	32 (52.46)	352 (58.67)	0.518
BMI, kg/m^2^	23.72 ± 3.14	23.44 ± 3.38	23.37 ± 3.04	23.51 ± 3.2	26.36 ± 3.43	< 0.001
Time from symptom onset to admission (h)	22 (8,68)	72 (12,160)	18 (4,50)	26 (9.8,94)	22 (8,98)	0.021
STEMI, n (%)	31 (77.5)	11 (73.33)	4 (66.67)	46 (75.41)	391 (65.17)	0.079
Anterior MI, n (%)	20 (50)	7 (46.67)	2 (33.33)	29 (47.54)	306 (51)	0.702
Heart rate, bpm	95.66 ± 16.72	104.21 ± 19.15	98.81 ± 20.04	99.33 ± 19.47	74.82 ± 16.2	< 0.001
SBP, mmHg	114.47 ± 22.54	109.23 ± 21.24	109.7320.94	111.03 ± 24.1	127.17 ± 23.08	0.045
KILLIP class						
KILLIP I, n (%)	8 (20)	1 (6.67)	0 (0)	9 (16.36)	338 (56.33)	< 0.001
KILLIP II, n (%)	20 (50)	9 (60)	2 (33.33)	31 (50.82)	201 (33.5)	< 0.001
KILLIP III, n (%)	6 (15)	3 (20)	3 (50)	12 (19.67)	35 (5.83)	0.002
KILLIP IV, n (%)	6 (15)	2 (13.3)	1 (16.67)	9 (14.75)	26 (4.33)	0.025
β-RB within 24 h, n (%)	12 (30)	6 (40)	2 (33.33)	20 (32.79)	303 (50.5)	0.071
ACEI/ARB within 24 h, n (%)	3 (7.5)	1 (6.67)	1 (16.67)	5 (8.2)	71 (11.83)	0.314
ESR, mm/h	24.28 ± 18.78	43.71 ± 29.7	37.4 ± 22.71	31.14 ± 23.48	12.15 ± 13.02	0.012
HBA1C, %	6.53 ± 1.01	6.39 ± 1.26	6.47 ± 1.11	6.44 ± 1.1	6.36 ± 0.98	0.102
BNP (pg/ml)	538.3 ± 304.63	1025.48 ± 406.22	849.06 ± 392.8	772.33 ± 368.09	274.78 ± 146.53	< 0.001
WBC, *10^9^/L	12.23 ± 4.79	11.64 ± 4.23	12.01 ± 4.49	12.11 ± 4.62	9.66 ± 3.27	< 0.001
RBC, *10^12^/L	4.06 ± 0.48	3.92 ± 0.51	3.99 ± 0.44	4.01 ± 0.39	4.36 ± 0.52	0.008
Hb, g/L	123.88 ± 14.19	120.43 ± 17.15	119.19 ± 16.2	121.47 ± 15.88	132.48 ± 17.49	0.007
D-dimer, mg/L FEU	1.39 ± 1.41	1.89 ± 1.46	1.37 ± 1.5	1.44 ± 1.33	1.11 ± 2.29	0.119
CK-MB, ng/ml	59.1 (11.74, 158.55)	34.7 (6.41, 132.2)	42.21 (5.29, 139.66)	48.18 (7.25, 151.77)	19.54 (4.07, 79.41)	0.009
CTnI, ng/ml	32.55 (6.71, 59.7)	8.49 (5.47, 31.86)	11.23 (4.78, 36.7)	22.61 (6.17, 44.42)	9.18 (2.73, 38.22)	0.014
LDL, mmol/L	2.5 ± 0.95	2.72 ± 0.81	2.52 ± 0.82	2.55 ± 0.95	2.57 ± 0.88	0.933
SCR, umol/L	100.48 ± 51.82	118.63 ± 44.29	104.22 ± 48.9	104.4 ± 48.7	91.4 ± 50.6	0.217
eGFR, ml/min/1.73 m^2^	69.01 ± 30.18	53.23 ± 21.42	62.31 ± 26.4	62.89 ± 25.3	82.37 ± 31.1	0.001
SUA, umol/L	329.8 ± 75.42	384.73 ± 97.18	352.41 ± 79.3	346.39 ± 85.75	337.1 ± 97.28	0.525
ABO						
A, n (%)	21 (52.5)	6 (40)	3 (50)	30 (49.18)	213 (35.5)	0.012
B, n (%)	11 (27.5)	5 (33.33)	2 (33.33)	18 (29.51)	227 (37.83)	0.213
O, n (%)	6 (15)	3 (20)	1 (16.67)	10 (16.39)	116 (19.33)	0.775
AB, n (%)	2 (5)	1 (6.67)	0 (0)	3 (4.92)	44 (7.33)	0.841
LVEDd, mm	48.3 ± 9.49	49.2 ± 7.08	48.41 ± 7.48	48.55 ± 8.91	48.36 ± 5.1	0.803
LVESd, mm	38.27 ± 10.16	36.76 ± 8.24	37.01 ± 8.79	37.47 ± 9.7	35.76 ± 7.12	0.389
LVEF (%)	48.12 ± 9.29	48.15 ± 10.57	47.78 ± 8.82	48.14 ± 10.28	58.19 ± 9.92	< 0.001
GRACE RS	197.29 ± 39.41	211.22 ± 40.19	201.4 ± 38.52	200.12 ± 41.73	161 ± 31.5	< 0.001
Primary PCI treatment, n (%)	14 (35)	4 (26.67)	3 (50)	21 (34.43)	416 (69.33)	< 0.001
In-hospital mortality, n (%)	38 (95)	10 (66.67)	2 (33.33)	50 (81.97)	25 (4.17)	< 0.001

Data are number (%), mean (SD), or median (IQR)

FWR, free wall rupture; VSR, ventricular septal rupture; PMR, papillary muscle rupture; HT, hypertension; DM, diabetes mellitus; CHF, chronic heart failure; TIA, transient ischemic attack; MI, myocardial infarction; CAD, coronary artery disease; PCI, percutaneous coronary intervention; CABG, coronary artery bypass grafting; AF, atrial fibrillation; BMI, body mass index; STEMI, ST-segment elevation myocardial infarction; SBP, systolic blood pressure; β-RB, β-receptor blocker; ACEI, angiotensin-converting enzyme inhibitor; ARB, agiotensin Receptor Blocker; ESR, erythrocyte sedimentation rate; HBA1C. glycosylated hemoglobin; BNP, brain natriuretic peptide; WBC, white blood cell; RBC, red blood cell; Hb, haemoglobin; CK-MB, creatine kinase MB; CTnI, cardiac troponin I; LDL-C, low-density lipoprotein cholesterol; SCR, serum creatinine; eGFR, estimated glomerular filtration rate; SUA, serum uric acid; LVEDd, left ventricular end-diastolic dimension; LVESd, left ventricular end- systolic dimension; LVEF, left ventricular ejection fraction; GRACE RS, The Global Registry of Acute Coronary Events risk score; pPCI, primary percutaneous coronary intervention

**Table 2:** Time from AMI onset to HR

Variables	Total (n = 61)	≤ 24 h (n = 32)	2–3 days (n = 8)	4–6 days (n = 18)	≥ 7 days (n = 3)
FWR, n (%)	40	21 (52.5)	5 (12.5)	12 (30)	2 (5)
VSR, n (%)	15	7 (46.67)	2 (13.33)	5 (33.33)	1 (6.67)
PMR, n (%)	6	4 (66.67)	1 (16.67)	1 (16.67)	0 (0)

AMI, acute myocardial infarction; HR, heart rupture; FWR, free wall rupture; VSR, ventricular septal rupture; PMR, papillary muscle rupture

### ABO blood groups and HR

Blood group B was most common (37.07%), followed by blood group A (36.76%), O (19.06%), and AB (7.11%) (Table 3). The frequency of blood group A was significantly higher in HR patients (49.18% vs. 35.5% in non-TM group, *P* = 0.012, Table 1). However, in the ANOVA test, HR events did not differ from 4 other blood groups (F = 2.086, *P* = 0.105). In multivariate logistic regression analysis, compared to non-A blood groups, blood group A remained an independent predictor for HR after AMI, after the adjustment for anthropometric variables such as age, gender, heart rate at admission, BMI and SBP (OR = 2.781, 95% CI 1.174–7.198, *P* = 0.035, Table 4 model 1). The association between blood group A and an elevated risk of HR after AMI was also observed in different multivariate regression models (*P* < 0.05, Table 4).

**Table 3:** Baseline characteristics according to ABO Blood Groups

Variables	A (n = 243)	B (n = 245)	O (n = 126)	AB (n = 47)	*P* (A vs non-A)
Age, years	70.7 ± 10.03	67.76 ± 9.56	69.68 ± 8.68	69.77 ± 9.08	0.952
Male, n (%)	166 (68.3)	178 (72.65)	78 (61.9)	31 (65.96)	0.696
HT, n (%)	168 (69.14)	173 (70.61)	83 (65.87)	35 (74.47)	0.88
DM, n (%)	66 (27.16)	71 (28.98)	16 (12.7)	7 (14.89)	0.645
CHF, n (%)	10 (4.12)	10 (4.08)	6 (4.76)	3 (6.38)	0.355
History of MI, n (%)	26 (10.7)	30 (12.24)	12 (9.52)	4 (8.51)	0.581
History of CAD, n (%)	45 (18.52)	53 (21.63)	21 (16.67)	8 (17.02)	0.814
History of PCI, n (%)	35 (14.4)	37 (15.1)	12 (9.52)	5 (10.64)	0.88
History of CABG, n (%)	6 (2.47)	6 (2.45)	2 (1.59)	1 (2.13)	0.661
Current smoker, n (%)	144 (59.26)	152 (62.04)	58 (46.03)	30 (63.83)	0.574
BMI, kg/m^2^	25.55 ± 3.76	24.25 ± 3.27	24.52 ± 2.58	25.39 ± 3.96	0.159
Symptom onset time (h)	38.92 ± 45.82	28.52 ± 43.67	33.61 ± 47.55	38.54 ± 47.27	0.762
STEMI, n (%)	165 (67.9)	174 (71.02)	69 (54.76)	29 (61.7)	0.382
Anterior MI, n (%)	122 (50.21)	131 (53.47)	59 (46.83)	23 (48.94)	
Heart rate, bpm	82.93 ± 18.56	80.91 ± 15.39	80.43 ± 19.96	73.23 ± 12.54	0.401
SBP, mmHg	127.33 ± 25.55	123.88 ± 23.06	125.57 ± 23.51	128.46 ± 20.68	0.609
DBP, mmHg	71.06 ± 12.95	73.38 ± 11.75	75.32 ± 11.26	69.85 ± 9.65	0.505
KILLIP class					
KILLIP I, n (%)	128 (52.67)	129 (52.65)	63 (50)	27 (57.45)	0.371
KILLIP II, n (%)	85 (34.98)	88 (35.92)	46 (36.51)	13 (26.66)	0.296
KILLIP III, n (%)	17 (7)	18 (7.35)	9 (7.14)	3 (6.38)	0.682
KILLIP IV, n (%)	13 (5.35)	10 (4.08)	8 (6.35)	4 (8.51)	0.267
β-RB in 24 h, n (%)	120 (49.38)	117 (47.76)	61 (48.41)	25 (53.19)	0.611
ACEI/ARB in 24 h, n (%)	29 (11.93)	26 (10.61)	15 (11.9)	6 (12.77)	0.68
ESR, mm/h	15.72 ± 14.52	18.17 ± 13.7	16.93 ± 13.81	13.69 ± 10	0.574
HBA1C, %	6.74 ± 1.25	6.49 ± 1.07	6.33 ± 1.3	6.19 ± 0.48	0.058
BNP (pg/ml)	760.25 (441.3, 1018.5)	641.9 (316.85, 1120)	694.22 (306.28, 994.18)	667.02 (285.95, 1010,6)	0.236
WBC, *10^9^/L	10.5 ± 4.17	10.36 ± 3.84	9.63 ± 3.93	9.36 ± 2.01	0.298
RBC, *10^12^/L	4.21 ± 0.9	4.14 ± 0.58	4.2 ± 0.48	4.02 ± 0.48	0.097
Hb, g/L	128.28 ± 22.41	127.59 ± 19.56	129.14 ± 15.23	124.33 ± 13.54	0.328
D-dimer, mg/L FEU	1.31 ± 1.21	1.37 ± 1.63	1.32 ± 0.65	1.11 ± 0.44	0.67
CK-MB, ng/ml	22 (5.95, 92.65)	28.9 (6.4, 127.03)	17.4 (1.9, 55.85)	12.1 (1.7, 43.85)	0.877
CTnI, ng/ml	7.53 (2.95, 33.7)	11.23 (6.01, 47.83)	5.62 (1.32, 39.65)	7.61 (1.32, 33.65)	0.308
LDL, mmol/L	2.58 ± 0.97	2.59 ± 0.79	2.33 ± 0.77	2.67 ± 0.95	0.15
SCR, umol/L	101.44 ± 59.81	103.9 ± 61.36	84.82 ± 21.95	76.5 ± 23.22	0.392
eGFR, ml/min/1.73 m^2^	71.7 ± 30.63	78.32 ± 36.61	79.41 ± 25.42	89.36 ± 34.77	0.521
SUA, umol/L	344.81 ± 101.99	338.04 ± 103.9	311.62 ± 87.29	317.36 ± 51.76	0.56
LVEDd, mm	48.31 ± 8	49.26 ± 6.33	46.91 ± 4.86	47.23 ± 5.22	0.23
LVESd, mm	35.93 ± 8.47	36.04 ± 8.09	33.61 ± 7.27	36.08 ± 8.22	0.48
LVEF (%)	52.09 ± 10.91	55.16 ± 11.6	58.43 ± 10.62	55.46 ± 11.36	0.923
GRACE RS	182.7 ± 42.85	174.03 ± 44.38	167.36 ± 36.23	169.08 ± 32.71	0.778
Primary PCI treatment, n (%)	161 (66.26)	166 (67.76)	80 (63.49)	30 (63.83)	0.668
Heart rupture, n (%)	30 (12.35)	18 (7.35)	10 (7.94)	3 (6.38)	< 0.001
Mortality, n (%)	33 (13.58)	25 (10.2)	12 (9.52)	5 (10.64)	0.024

Data are number (%), mean (SD), or median (IQR)

HT, hypertension; DM, diabetes mellitus; CHF, chronic heart failure; TIA, transient ischemic attack; MI, myocardial infarction; CAD, Coronary artery disease; PCI, percutaneous coronary intervention; CABG, coronary artery bypass grafting; AF, atrial fibrillation; BMI, body mass Index; STEMI, ST-segment elevation myocardial infarction; SBP, systolic blood pressure; β-RB, β-receptor blocker; ACEI, angiotensin-converting enzyme inhibitor; ARB, agiotensin Receptor Blocker; ESR, erythrocyte sedimentation rate; HBA1C. glycosylated hemoglobin; BNP, brain natriuretic peptide; WBC, white blood cell; RBC, red blood cell; Hb, haemoglobin; CK-MB, creatine kinase MB; CTnI, cardiac troponin I; LDL-C, low-density lipoprotein cholesterol; LP(a), Lipoprotein (a); SCR, serum creatinine; eGFR, estimated glomerular filtration rate; SUA, serum uric acid; LVEDd, left ventricular end-diastolic dimension; LVESd, left ventricular end- systolic dimension; LVEF, Left ventricular ejection fraction; GRACE RS, The Global Registry of Acute Coronary Events risk score; PCI, percutaneous coronary intervention

**Table 4:** Multiple logistic regression analysis for the association between ABO blood groups and HR after AMI

	β	OR (95% CI)	*P* value
Model 1			
*Blood group A*	*1.023*	*2.781 (1.174–7.198)*	*0.035*
Age, years	1.68	4.397 (1.698–11.578)	0.001
Female	0.13	1.139 (1.065–1.218)	< 0.001
Heart rate at admission, bpm	0.053	1.054 (1.023–1.086)	0.001
BMI, kg/m^2^	− 0.127	0.881 (0.764–1.015)	0.079
SBP, mmHg	− 0.013	0.987 (0.969, 1.006)	0.179
Model 2			
*Blood group A*	*0.895*	*2.448 (1.121–5.869)*	*0.045*
Age, years	0.803	2.232 (0.913–5.445)	0.78
Female	0.171	1.187 (1.102–1.278)	< 0.001
ESR, mm/h	0.051	1.053 (1.017–1.09)	0.003
BNP, pg/ml	0.01	1.011 (0.997–1.019)	0.853
CTnI, ng/ml	0.007	1.007 (0.999–1.015)	0.088
Model 3			
*Blood group A*	*0.697*	*2.107 (1.065–4.568)*	*0.046*
Age, years	1.324	3.757 (1.548–9.12)	0.003
Female	0.129	1.138 (1.065–1.215)	0.001
LVEF, %	− 0.064	0.938 (0.902–0.977)	0.002
Model 4			
*Blood group A*	*0.73*	*2.075 (1.002–4.288)*	*0.048*
Age, years	0.863	2.371 (1.035–5.433)	0.031
Female	0.132	1.141 (1.07–1.217)	0.002
No pPCI treatment	1.072	2.928 (1.418–7.344)	0.005
Model 5			
*Blood group A*	*0.687*	*2.212 (1.064–5.168)*	*0.039*
GRACE RS	0.019	1.02 (1.01–1.029)	< 0.001

Italicized value indicates that blood group A was significantly associated with an elevated risk of HR after AMI in different models

HR, heart rupture; AMI, acute myocardial infarction; OR, odds ratio; CI, Confidence interval; BMI, body mass index; SBP, systolic blood pressure; ESR, erythrocyte sedimentation rate; BNP, brain natriuretic peptide; CTnI, cardiac troponin I; LVEF, left ventricular ejection fraction; pPCI, primary primary percutaneous coronary intervention; GRACE RS, Global Registry of Acute Coronary Events risk score

Variables included in model 1 are blood group A, age, female gender, heart rate at admission, BMI and SBP

Variables included in model 2 are blood group A, age, female gender, ESR, BNP and CTnI

Variables included in model 3 are blood group A, age, female gender and LVEF

Variables included in model 4 are blood group A, age, female gender and no pPCI treatment

Variables included in model 5 are blood group A and the GRACE RS

## Discussion

In the present study, A significant association was observed between blood group A and an increased risk of HR after AMI, in both univariate and multivariate analyses. As far as we know, this is the first study to reveal that blood group A is an independent risk factor for HR in Chinese AMI patients.

HR was still one of the most serious complications after AMI, even with the worldwide use of PCI or some other modern therapies [1–3, 26]. In the pre- reperfusion era, HR occurred in about 6% of all admitted AMI patients [2, 5].

The incidence of HR after AMI is less than 1% reported in modern studies and similar results can be seen in the present study (0.75%) [1, 27, 28]. HR, especially FWR, is known as a desperate complication after AMI. The in-hospital mortality of HR patient remains very high in spite of the rapid advances in diagnostic and treatment methods. In this study, the in-hospital death rate of patients with HR was 81.97%, with 95%, 66.67% and 33.33% in FWR, VSR and PMR patients, respectively. Similar or a little lower hospital mortality rates have been reported in previous studies [7, 29–31].

The antigens of ABO blood groups are mainly expressed on the surface of red blood cells (RBC), and are also expressed on vascular endothelium, gastrointestinal, oral and bronchopulmonary epithelium, platelets (PLT) and neurocytes [10, 18, 32]. There is conflicting data about the association between ABO blood groups and CAD. A number of studies have proved the important role of the ABO blood groups in the prognosis of CAD patients. Carpeggiani et al. [11] showed that blood group A is associated with increased mortality in patients with CAD, particularly in younger females. Cetin et al. [18] reported that ABO blood groups were determined to be significant prognostic indicators of short and long-term cardiovascular adverse events and mortality in patients with STEMI undergoing pPCI. A study by Ketch et al. [16] showed that compared to blood group O, patients with non-O blood groups have larger infarct sizes but similar 1 year outcomes. However, the association between ABO blood groups and CAD or cardiovascular risk factors had not been confirmed by some other studies [14, 33, 34]. None of these previous studies demonstrated the association between ABO blood groups and HR after AMI, the current study was designed to provide evidence.

The underlying mechanisms through which ABO blood groups may participate in the pathogenesis of HR after AMI remain unclear. Patients with non-O compared to O blood group have more myocardial necrosis, larger myocardial infarct size and reduced pre-procedural thrombolysis in myocardial infarction (TIMI) flow of coronary, accounting for the higher level of von Willebrand factor (VWF) and factor VIII in non-O blood groups, especially in A and B blood groups [16, 17, 21]. This may be one potential reason for the increased HR risk in AMI patients with blood group A. Higher CTnI and lower LVEF are two major clinical indicators related to a larger myocardial infarct size [35]. In our study, however, there was no statistical difference in CTnI or LVEF between blood group A and other blood groups. The bias caused by small sample size of our study may be responsible for this. However, whether blood type A increases the risk of HR after AMI by causing a larger myocardial infarct size should be further studied in larger cohort with the help of cardiac magnetic resonance. Moreover, genome-wide association studies (GWAS) have identified that ABO blood groups gene as a locus for diabetes mellitus and many inflammatory biomarkers, such as IL-10, soluble E-selectin, P-selectin and intercellular adhesive molecule 1 (ICAM-1) [17, 36, 37]. Therefore, blood group antigens may increase thrombus burden and inflammatory substances level in circulation, which can increase the risk of HR [37, 38].

HR were more prevalent in older and female patients. Longer Time from symptom onset to admission, higher heart rate at admission, KILLIP class, BNP, ESR, WBC, CTNI, CK-MB and blood group A, were also seen in patients with HR after AMI. Moreover, HR group had significantly lower BMI, RBC, Hb, LVEF, eGFR, and had lower chance to receive pPCI treatment. Most of these HR related factors, such as age, female gender, BNP, heart rate at admission and no pPCI treatment, have also been reported previously, except blood group A [21, 26, 30, 39, 40]. According to the principle of 10 outcome events per variable, multivariate logistic regression analyses included less than 6 variables was conducted [41]. After adjusting anthropometric risk factors of HR (age, gender, heart rate at admission, BMI and SBP), blood group A was associated with HR after AMI independently (OR = 2.781, 95% CI 1.174–7.198, *P* = 0.035). We then conducted another multivariate logistic regression model that included age, gender and blood biomarkers related to HR (ESR, BNP and CTnI). After adjusting these variables, blood group A remained as an independent predictor for HR after AMI (OR = 2.488, 95% CI 1.121–5.869, *P* = 0.045). The association between blood group A and an elevated risk of HR after AMI was also observed in different multivariate regression models that included echocardiographic index (LVEF) and treatment strategy (received pPCI treatment or not).

The GRACE RS has been recognized as a validated tool to predict mortality risk of ACS patients and it has been recommend by current clinical guidelines [42–45]. The value of the GRACE RS in predicting HR after AMI was rarely reported, and might be able to predict HR [21]. The GRACE RS of patients with HR was significantly higher than patients without HR (199.14 ± 41.03 vs 164 ± 36.54, *P* < 0.001).We then conducted a logistic regression analysis that included the GRACE RS as an independent variable, the analysis also indicated that blood group A is a risk factor of HR (OR = 2.212, 95% CI 1.064–5.168, *P* = 0.039). To the best of our knowledge, this is the first study proving the association between ABO blood groups and HR after AMI.

There were 3 limitations to the current study. First, this a single-center research with a relatively small population. Second, as a retrospectively case–control study, the potential cause-effect relationship was unknown. Finally, we only observed an independent association between blood group A and HR after AMI, the underlying mechanisms should be studied in the future.

## Conclusion

Blood group A is an independent risk factor for HR in Chinese AMI patients. Evaluation of this parameter may help with risk stratification of HR in AMI patients.

## Data Availability

The datasets generated and/or analysed during the current study are not publicly available due to the items of the informed consent forms of our study and the restrictions by the Beijing Chaoyang Hospital, even an anonymised version of the dataset could not be made available either. The authors used the dataset under an agreement with the Beijing Chaoyang Hospital for the present study.

## Abbreviations

CAD: Coronary artery disease
HR: Heart rupture
AMI: Acute myocardial infarction
BMI: Body mass index
SBP: Systolic blood pressure
FWR: Free wall rupture
VSR: Ventricular septal rupture
PMR: Papillary muscle rupture
MR: Mitral regurgitation
STEMI: ST-segment elevation myocardial infarction
NSTEMI: Non- ST-segment elevation myocardial infarction
CTnI: Cardiac troponin-I
ECG: Electrocardiogram
LBBB: Left bundle branch block
eGFR: Estimated glomerular filtration rate
MDRD: Modification of Diet in Renal Disease
GRACE RS: Global Registry of Acute Coronary Events risk score
ACS: Acute coronary syndromes
pPCI: Primary percutaneous coronary intervention
CABG: Coronary artery bypass grafting
RBC: Red blood cells
PLT: Platelets
TIMI: Thrombolysis in myocardial infarction
VWF: Von Willebrand factor
GWAS: Genome-wide association studies
ICAM-1: Intercellular adhesive molecule 1

## References

[1] Becker RC, Charlesworth A, Wilcox RG, Hampton J, Skene A, Gore JM (1995). Cardiac rupture associated with thrombolytic therapy: Impact of time to treatment in the late assessment of thrombolytic efficacy (LATE) study. J Am Coll Cardiol.

[2] Figueras J, Alcalde O, Barrabes JA, Serra V, Alguersuari J, Cortadellas J (2008). Changes in hospital mortality rates in 425 patients with acute ST-elevation myocardial infarction and cardiac rupture over a 30-year period. Circulation.

[3] Becker RC, Gore JM, Lambrew C, Douglas Weaver W, Michael Rubison R, French WJ (1996). A composite view of cardiac rupture in the United States national registry of myocardial infarction. J Am Coll Cardiol.

[4] López-Sendón J, González A, de Sá EL, Coma-Canella I, Roldán I, Domínguez F (1992). Diagnosis of subacute ventricular wall rupture after acute myocardial infarction: sensitivity and specificity of clinical, hemodynamic and echocardiographic criteria. J Am Coll Cardiol.

[5] Slater J, Brown RJ, Antonelli TA, Menon V, Boland J, Col J (2000). Cardiogenic shock due to cardiac free-wall rupture or tamponade after acute myocardial infarction: a report from the SHOCK Trial Registry. J Am Coll Cardiol.

[6] Pohjola-Sintonen SMJ, Stone PH, Willich SN, Antman EM, Davis VG, Parker CB, Braunwald E (1989). Ventricular septal and free wall rupture complicating acute myocardial infarction: experience in the multicenter investigation of limitation of infarct size. Am Heart J.

[7] Bajaj A, Sethi A, Rathor P, Suppogu N, Sethi A (2015). Acute complications of myocardial infarction in the current era: diagnosis and management. J Investig Med.

[8] Moreno R, López-Sendón J, García E, de Isla LP, de Sá EL, Ortega A (2002). Primary angioplasty reduces the risk of left ventricular free wall rupture compared with thrombolysis in patients with acute myocardial infarction. J Am Coll Cardiol.

[9] Leroux É, Chauvette V, Voisine P, Dagenais F, Charbonneau É, Beaudoin J, Dubois-Sénéchal É, Dubois M, Sénéchal M (2019). Clinical and echocardiographic presentation of postmyocardial infarction papillary muscle rupture. Echocardiography.

[10] Vasan SK, Rostgaard K, Majeed A, Ullum H, Titlestad KE, Pedersen OB (2016). ABO blood group and risk of thromboembolic and arterial disease: a study of 15 million blood donors. Circulation.

[11] Carpeggiani C, Coceani M, Landi P, Michelassi C, L'Abbate A (2010). ABO blood group alleles: a risk factor for coronary artery disease. An angiographic study. Atherosclerosis.

[12] Lee H-F, Lin Y-C, Lin C-P, Wang C-L, Chang C-J, Hsu L-A (2012). Association of blood group A with coronary artery disease in young adults in Taiwan. Intern Med.

[13] Tufano A, Coppola A, Nardo A, Bonfanti C, Crestani S, Cerbone AM (2013). Non-O blood group as a risk factor for cerebral vein thrombosis. Thromb Haemost.

[14] Gong P, Luo SH, Li XL, Guo YL, Zhu CG, Xu RX (2014). Relation of ABO blood groups to the severity of coronary atherosclerosis: an Gensini score assessment. Atherosclerosis.

[15] Hajizadeh R, Kavandi H, Nadiri M, Ghaffari S (2016). Association of ABO blood group with incidence and outcome of acute pulmonary embolism. Turk Kardiyol Dern Ars.

[16] Ketch TR, Turner SJ, Sacrinty MT, Lingle KC, Applegate RJ, Kutcher MA (2008). ABO blood types: influence on infarct size, procedural characteristics and prognosis. Thromb Res.

[17] Johansson A, Alfredsson J, Eriksson N, Wallentin L, Siegbahn A (2015). Genome-wide association study identifies that the ABO blood group system influences interleukin-10 levels and the risk of clinical events in patients with acute coronary syndrome. PLoS ONE.

[18] Cetin MS, Ozcan Cetin EH, Aras D, Topaloglu S, Temizhan A, Kisacik HL (2015). Non-O blood groups can be a prognostic marker of in-hospital and long-term major adverse cardiovascular events in patients with ST elevation myocardial infarction undergoing primary percutaneous coronary intervention. Thromb Res.

[19] Fu Y, Li KB, Yang XC (2019). A risk score model for predicting cardiac rupture after acute myocardial infarction. Chin Med J (Engl).

[20] Kolte D, Khera S, Aronow WS, Mujib M, Palaniswamy C, Sule S (2014). Trends in incidence, management, and outcomes of cardiogenic shock complicating ST-elevation myocardial infarction in the United States. J Am Heart Assoc.

[21] López-Sendón J, Gurfinkel E, Lopez de Sa E, Agnelli G, Gore J, Steg P (2010). Factors related to heart rupture in acute coronary syndromes in the Global Registry of Acute Coronary Events. Eur Heart J.

[22] Ma YC, Zuo L, Chen JH, Luo Q, Yu XQ, Li Y (2006). Modified glomerular filtration rate estimating equation for Chinese patients with chronic kidney disease. J Am Soc Nephrol.

[23] Fox KA, Anderson FA, Dabbous OH, Steg PG, Lopez-Sendon J, Van de Werf F (2007). Intervention in acute coronary syndromes: do patients undergo intervention on the basis of their risk characteristics? The Global Registry of Acute Coronary Events (GRACE). Heart.

[24] Fox KA, Dabbous OH, Goldberg RJ, Pieper KS, Eagle KA, Van de Werf F (2006). Prediction of risk of death and myocardial infarction in the six months after presentation with acute coronary syndrome: prospective multinational observational study (GRACE). BMJ.

[25] Tang EW, Wong C-K, Herbison P (2007). Global Registry of Acute Coronary Events (GRACE) hospital discharge risk score accurately predicts long-term mortality post acute coronary syndrome. Am Heart J.

[26] Gueret P, Khalife K, Jobic Y, Fillipi E, Isaaz K, Tassan-Mangina S (2008). Echocardiographic assessment of the incidence of mechanical complications during the early phase of myocardial infarction in the reperfusion era: a French multicentre prospective registry. Arch Cardiovasc Dis.

[27] Fonarow GC, Wright RS, Spencer FA, Fredrick PD, Dong W, Every N (2005). Effect of statin use within the first 24 hours of admission for acute myocardial infarction on early morbidity and mortality. Am J Cardiol.

[28] Pujol E, Morales M, Roelandt JR, Perez MJ, Masia R, Sala J (2008). Partial ventricular septal defect (Pacman heart). Eur J Echocardiogr.

[29] Nozoe M, Sakamoto T, Taguchi E, Miyamoto S, Fukunaga T, Nakao K (2014). Clinical manifestation of early phase left ventricular rupture complicating acute myocardial infarction in the primary PCI era. J Cardiol.

[30] Fazlinezhad A, Rezaeian MK, Yousefzadeh H, Ghaffarzadegan K, Khajedaluee M (2011). Plasma brain natriuretic peptide (BNP) as an indicator of left ventricular function, early outcome and mechanical complications after acute myocardial infarction. Clin Med Insights Cardiol.

[31] Sakaguchi G, Komiya T, Tamura N, Kobayashi T (2008). Surgical treatment for postinfarction left ventricular free wall rupture. Ann Thorac Surg.

[32] Zouine S, Marnissi F, Otmani N, Bennani Othmani M, El Wafi M, Kojok K (2016). ABO blood groups in relation to breast carcinoma incidence and associated prognostic factors in Moroccan women. Med Oncol.

[33] Amirzadegan A, Salarifar M, Sadeghian S, Davoodi G, Darabian C, Goodarzynejad H (2006). Correlation between ABO blood groups, major risk factors, and coronary artery disease. Int J Cardiol.

[34] Biancari F, Satta J, Pokela R, Juvonen T (2002). ABO blood group distribution and severity of coronary artery disease among patients undergoing coronary artery bypass surgery in Northern Finland. Thromb Res.

[35] Di Chiara A, Dall'Armellina E, Badano LP, Meduri S, Pezzutto N, Fioretti PM (2010). Predictive value of cardiac troponin-I compared to creatine kinase-myocardial band for the assessment of infarct size as measured by cardiac magnetic resonance. J Cardiovasc Med (Hagerstown).

[36] Paterson AD, Lopes-Virella MF, Waggott D, Boright AP, Hosseini SM, Carter RE, Shen E, Mirea L, Bharaj B, Sun L, Bull SB, Diabetes Control and Complications Trial/Epidemiology of Diabetes Interventions and Complications Research Group. Genome-wide association identifies the ABO blood group as a major locus associated with serum levels of soluble E-selectin. Arterioscler Thromb Vasc Biol 2009;29(11):1958–67. 10.1161/ATVBAHA 10.1161/ATVBAHA.109.192971 PMC3147250 19729612

[37] Barbalic M, Dupuis J, Dehghan A, Bis JC, Hoogeveen RC, Schnabel RB (2010). Large-scale genomic studies reveal central role of ABO in sP-selectin and sICAM-1 levels. Hum Mol Genet.

[38] Investigators MRS (2017). Aortic wall inflammation predicts abdominal aortic aneurysm expansion, rupture, and need for surgical repair. Circulation.

[39] Pohjola-Sintonen S, Muller JE, Stone PH, Willich SN, Antman EM, Davis VG (1989). Ventricular septal and free wall rupture complicating acute myocardial infarction: experience in the Multicenter Investigation of Limitation of Infarct Size. Am Heart J.

[40] Honda S, Asaumi Y, Yamane T, Nagai T, Miyagi T, Noguchi T (2014). Trends in the clinical and pathological characteristics of cardiac rupture in patients with acute myocardial infarction over 35 years. J Am Heart Assoc.

[41] Steyerberg EW, Eijkemans MJ, Harrell FE, Habbema JD (2001). Prognostic modeling with logistic regression analysis: in search of a sensible strategy in small data sets. Med Decis Making.

[42] Amsterdam EA, Wenger NK, Brindis RG, Casey DE, Ganiats TG, Holmes DR (2014). 2014 AHA/ACC guideline for the management of patients with non–ST-elevation acute coronary syndromes: executive summary. J Am Coll Cardiol.

[43] Antman EM, Anbe DT, Armstrong PW, Bates ER, Green LA, Hand M (2004). ACC/AHA guidelines for the management of patients with ST-elevation myocardial infarction—executive summary. J Am Coll Cardiol.

[44] Anderson JL, Adams CD, Antman EM, Bridges CR, Califf RM, Casey DE (2013). 2012 ACCF/AHA focused update incorporated into the ACCF/AHA 2007 guidelines for the management of patients with unstable angina/non-ST-elevation myocardial infarction: a report of the American College of Cardiology Foundation/American Heart Association Task Force on Practice Guidelines. J Am Coll Cardiol.

[45] Hamm CW, Bassand JP, Agewall S, Bax J, Boersma E, Bueno H (2011). ESC Guidelines for the management of acute coronary syndromes in patients presenting without persistent ST-segment elevation: The Task Force for the management of acute coronary syndromes (ACS) in patients presenting without persistent ST-segment elevation of the European Society of Cardiology (ESC). Eur Heart J.

